# Gefitinib Induces Epidermal Growth Factor Receptor Dimers Which Alters the Interaction Characteristics with ^125^I-EGF

**DOI:** 10.1371/journal.pone.0024739

**Published:** 2011-09-12

**Authors:** Hanna Björkelund, Lars Gedda, Pavel Barta, Magnus Malmqvist, Karl Andersson

**Affiliations:** 1 Biomedical Radiation Sciences, Department of Radiology, Oncology and Radiation Science, Uppsala University, Uppsala, Sweden; 2 Ridgeview Instruments AB, Uppsala, Sweden; 3 Department of Pharmacology and Toxicology, Faculty of Pharmacy in Hradec Kralove, Charles University in Prague, Prague, Czech Republic; 4 Bioventia AB, Uppsala, Sweden; Hungarian Academy of Sciences, Hungary

## Abstract

The tyrosine kinase inhibitor gefitinib inhibits growth in some tumor types by targeting the epidermal growth factor receptor (EGFR). Previous studies show that the affinity of the EGF-EGFR interaction varies between hosting cell line, and that gefitinib increases the affinity for some cell lines. In this paper, we investigate possible mechanisms behind these observations. Real-time interaction analysis in LigandTracer® Grey revealed that the HER2 dimerization preventing antibody pertuzumab clearly modified the binding of ^125^I-EGF to EGFR on HER2 overexpressing SKOV3 cells in the presence of gefitinib. Pertuzumab did not affect the binding on A431 cells, which express low levels of HER2. Cross-linking measurements showed that gefitinib increased the amount of EGFR dimers 3.0–3.8 times in A431 cells in the absence of EGF. In EGF stimulated SKOV3 cells the amount of EGFR dimers increased 1.8–2.2 times by gefitinib, but this effect was cancelled by pertuzumab. Gefitinib treatment did not alter the number of EGFR or HER2 expressed in tumor cell lines A431, U343, SKOV3 and SKBR3. Real-time binding traces were further analyzed in a novel tool, Interaction Map, which deciphered the different components of the measured interaction and supports EGF binding to multiple binding sites. EGFR and HER2 expression affect the levels of EGFR monomers, homodimers and heterodimers and EGF binds to the various monomeric/dimeric forms of EGFR with unique binding properties. Taken together, we conclude that dimerization explains the varying affinity of EGF – EGFR in different cells, and we propose that gefitinib induces EGFR dimmers, which alters the interaction characteristics with ^125^I-EGF.

## Introduction

The extracellular binding of EGF to EGFR (also denoted ErbB1) triggers signals that are transduced through the cells and causes cell proliferation. Atypical activity and over-expression of EGFR is associated with a number of tumors, making it a common target for cancer research. Although well studied, many questions still remain unanswered about the interaction of EGF with EGFR, the resulting signaling and its involvement in cancer.

Apart from EGFR, the epidermal growth factor receptor family consists of the HER2, HER3 and HER4 receptors. The four receptors are known to dimerize, as homodimers or as heterodimers with other members of the family. The presence of EGFR dimers in EGF unstimulated cells have been discussed for many years, where some groups claim that EGFR dimers exist without stimulation [1], [2] while the more common belief is that EGFR dimerization requires a conformation change caused by the binding of EGF to monomeric EGFR [3], [4], [5]. Upon receptor dimerization, the cytoplasmic tyrosine kinase domain is activated through phosporylation [6]. HER2 is consecutively and ligand independently activated and is the preferred binding partner of EGFR [7]. HER2 dimerization with EGFR enhances and prolongs the downstream signaling caused by EGF binding [8], possibly due to the endocytosis deficiency of HER2, which in turn negatively affects the EGFR downregulation [9].

The EGF – EGFR interaction is known to be complex. Scatchard plots depict the presence of both low affinity and high affinity receptor populations, the latter less abundant [10], [11]. More recent studies presents a difference in affinity to EGF between the monomeric and homodimeric form of EGFR [12], where homodimers bind EGF more strongly. Furthermore, the high affinity population has been pointed out as the primary mediators of the EGFR signaling [13]. This vaguely suggests that EGFR dimers correspond to the high affinity population observed in the past.

The use of tyrosine kinase inhibitors (TKI) is a mean of disrupting the proliferation and anti-apoptotic downstream signaling [14]. One example is gefitinib (IRESSA™) which is directed towards EGFR and used as therapy against non-small cell lung cancer [15]. The aim is to inhibit tumor growth, but the response varies to a large extent between patients [16]. The reason behind this variation has been extensively investigated and discussed during the last decade, without much success. Lapatinib is another TKI targeted towards EGFR and HER2 for HER2 overexpressing mammary tumors [17]. It binds the inactive forms of EGFR as opposed to gefitinib that stabilize the active conformation [18], [19], [20], [21]. Gefitinib, but not lapatanib, have previously been shown to promote dimerization of EGFR. These dimers are considered non-active and conformationally different from ligand triggered dimer forms [21].

We have previously investigated the kinetic properties and the affinity of the ^125^I-EGF – EGFR interaction in different cell lines exposed to various treatments (complete or serum free medium, in the presence or absence of gefitinib) [22]. No effect of gefitinib was observed in U343 and SKBR3 cells, but it clearly affected the binding of ^125^I-EGF in A431 and SKOV3 cells. The overall affinity increased in both cell lines, but the kinetics properties (association and dissociation rate) were differently affected between the two. In addition, the shape of the real-time binding curves produced in LigandTracer Grey displayed evidence of more than one EGF binding interaction taking place simultaneously. The overall affinity was quantified and it was found to vary as much as 40 times between cell lines (from 0.2 nM in SKBR3 to 8 nM in A431).

The aim of this work was to investigate the biological mechanism behind the effect of gefitinib on the ^125^I-EGF – EGFR interaction and the differences in affinity between hosting cell lines. We present a detailed interaction analysis study of how the binding of ^125^I-EGF to EGFR is affected by tyrosine kinase inhibitors and the HER2 dimerization preventing antibody pertuzumab. Real-time binding traces of the interaction together with cross-linking assays show a connection between the effect of gefitinib and EGFR dimer levels. We also introduce a novel tool for binding trace analysis of complex interactions: the Interaction Map. Having previously been applied to molecular interaction analysis by SPR [23], this tool shows promise for resolving the complex results of protein-cell interactions.

## Results

### Gefitinib and serum free medium does not alter the EGFR or HER2 expression in A431, U343, SKOV3 or SKBR3 cells

The EGFR and HER2 expression level under different culturing conditions (complete medium or serum free medium, with or without gefitinib) was quantified either manually or with the kinetic extrapolation (KEX) method [24] using LigandTracer (Table 1). No clear differences were observed between treatments in any of the four cell lines. The apparent affinity to ^125^I-EGF varied greatly between the cell lines, as described previously [22] and presented in Table 1. For this set of cell lines, there seems to be some correlation between HER2 and EGFR expression and the overall affinity, with a higher affinity in cells expressing a large number of HER2 receptors and fewer EGFR.

**Table 1:** The EGFR and HER2 expression of A431, U343, SKOV3 and SKBR3 cells in four culturing conditions.

	A431			U343			SKOV3			SKBR3		
	EGFR	HER2	K_D, app_	EGFR	HER2	K_D, app_	EGFR	HER2	K_D, app_	EGFR	HER2	K_D, app_
Complete medium	2.1±0.4E6	1.5±0.1E5	8 nM	6.4±0.5E5	3.1±0.6E4	1.4 nM	3.4±0.6E5	2.0±0.3E7	0.9 nM	4.1±0.3E5	5.8±0.5E6	0.2 nM
Serum free medium	2.2±0.5E6	1.7±0.2E5		6.7±0.6E5	3.5±0.4E4		1.9±0.2E5	2.1±0.05E7		3.5±0.5E5	7.2±0.6E6	
Gefitinib +complete medium	2.2±0.3E6	2.6±0.5E5		6.9±1.0E5	2.2±0.3E4		3.2±0.6E5	2.3±0.1E7		4.6±0.6E5	6.4±2.6E6	
Gefitinib +serum free medium	1.3±0.2E6	2.0±0.5E5		5.3±0.1E5	2.7±0.3E4		2.18E+05E5	3.0±0.4E6		5.7±0.3E5	6.5±0.3E6	

Estimation of EGFR and HER2 receptors were conducted either manually or by the KEX method using LigandTracer. Estimations of the affinities have been described previously [22]. Data are presented as mean±S.E (n = 2−6). No clear effects of the treatments on the EGFR and HER2 expression is observed in either of the cell lines.

### Pertuzumab affects the binding of ^125^I-EGF to SKOV3 cells but not to A431 or U343 cells

**Figure 1:**
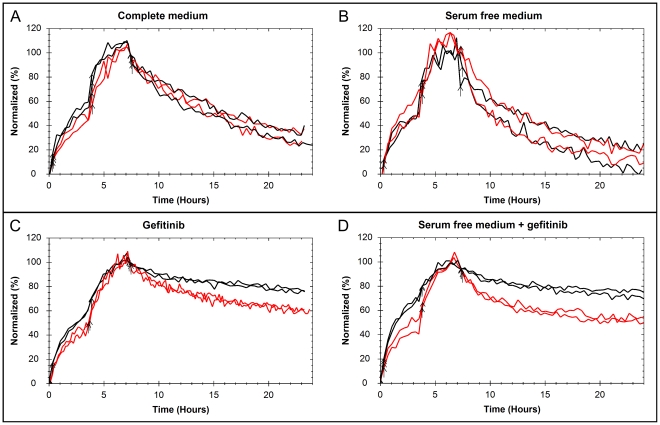
The effect of pertuzumab on the ^125^I-EGF – SKOV3 interaction. The binding of ^125^I-EGF to cultured SKOV3 cells in LigandTracer Grey, in the presence (red) or absence (black) of the HER2 dimerization disrupting antibody pertuzumab. The cells were cultured in A) complete medium, B) serum-free medium, C) complete medium containing 1 µM gefitinib or D) serum free medium with 1 µM gefitinib. The ^125^I-EGF – SKOV3 interaction is affected by pertuzumab, most evidently in gefitinib treated cells where the shape of the binding traces is clearly altered. The curves are representative data from triplicates or quadruplicates and have been normalized to the equilibration level of the second concentration for better comparison.

The dimerization preventing antibody pertuzumab binding to HER2 was added to investigate the role of EGFR-HER2 heterodimerization to the measured ^125^I-EGF interaction, producing binding traces presented as representative curves in Figure 1. Little or no differences were observed for U343 cells and A431 cells (data not shown). The addition of pertuzumab to SKOV3 cells modified the binding of ^125^I-EGF, observed as differences in curvature, when simultaneously treated with gefitinib. The increase in affinity caused by gefitinib, observed as a higher saturation of receptors with the first concentration and a slower dissociation (Fig. 1C and D, black curves), was essentially canceled by pertuzumab (Fig. 1C and D, red curves).

### The effects of EGF, gefitinib and pertuzumab on EGFR dimerization in A431 and SKOV3 cells

**Figure 2:**
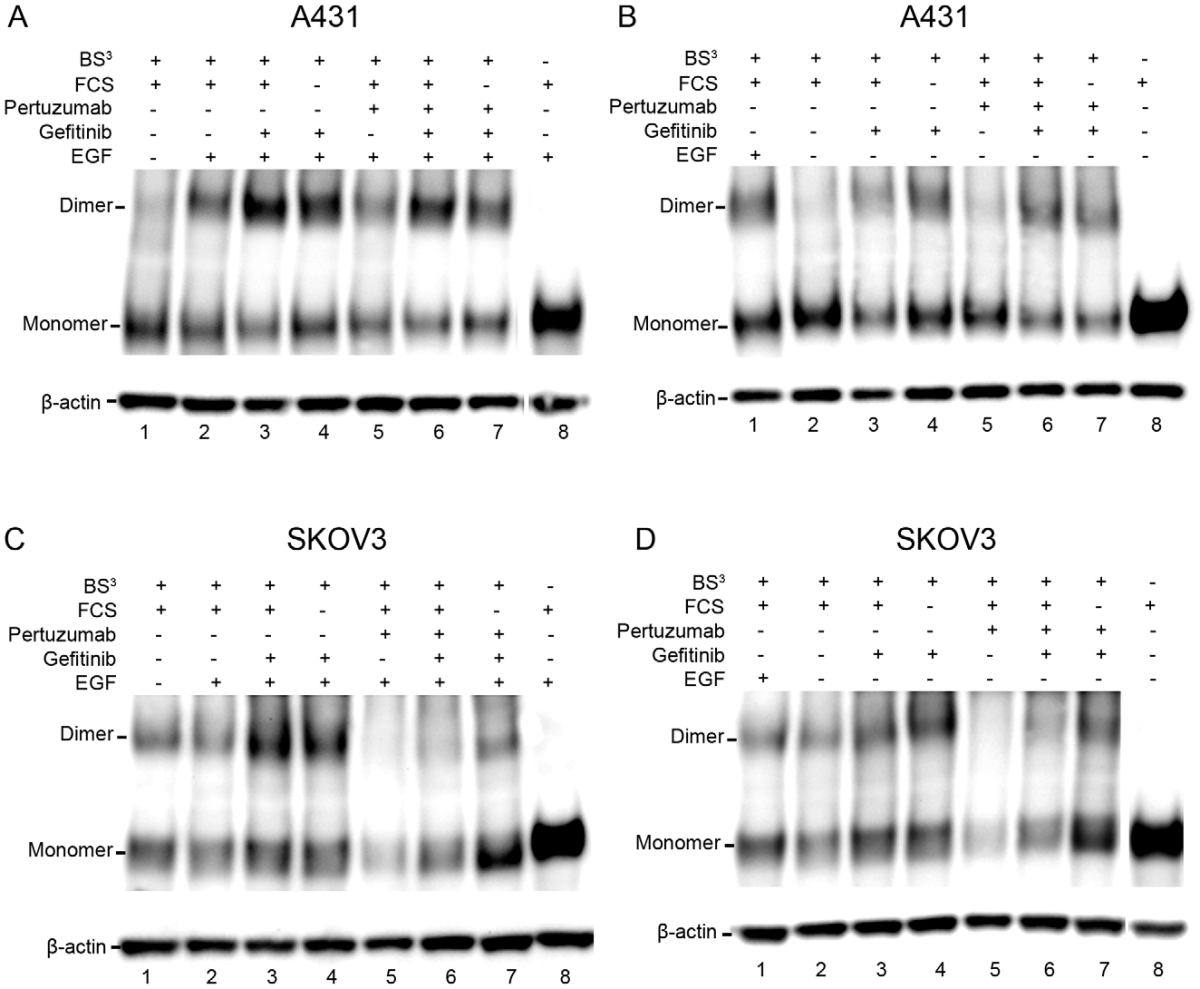
EGFR dimerization effects of EGF, gefitinib and pertuzumab observed in cross-linking analysis. EGFR targeting antibodies were used to depict EGFR monomers and dimers on A431 (A and B) and SKOV3 (C and D) lysates. The cells were treated with or without gefitinib and pertuzumab in complete (+FCS) or serum free medium. The difference between A and B as well as C and D is the stimulation of EGF. EGFR dimers exist without stimulation of EGF (A–D, lane 1–2), particularly in SKOV3 cells. Gefitinib increases the dimer levels in both cell lines, regardless of other treatment (A–D, lane 3–4 and 6–7). Pertuzumab does not affect the overall dimer levels of EGFR in A431 cells much (A and B, lane 5–7), but clearly disrupts EGFR dimerization in SKOV3 (C and D, lane 5–7). The BS^3^ free lysates were used as negative controls for the cross-linking assay and the β-actin band was used as a loading control. This is a representative of one of four experiments.

The amount of dimerized EGFR receptors in A431 and SKOV3 cells was studied by immunoblots using the reagent BS^3^ as a receptorcross-linking agent. The cells were incubated with or without EGF, fetal calf serum (FCS), gefitinib and pertuzumab before lysis, as presented in Figure 2 (representative data from one of four experiments). A presence of EGFR dimers without stimulation of EGF is seen in both cell lines, although to a higher extent in SKOV3 where the differences between stimulated and unstimulated cells are small (Fig. 2A–D, lane 1 and 2). No differences were observed between cells stimulated with complete or serum free medium in the presence of gefitinib (Fig. 2A–D, lane 3–4 and 6–7). Detection of β-actin was used as a loading control. However, the absolute number of EGFR seemed to vary between treatments.

The intensities of the EGFR monomer and dimer bands were quantified using ImageJ to study the effect of X (where X was EGF, gefitinib or pertuzumab) on dimerization. The effect was calculated as



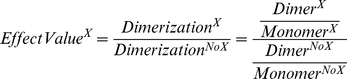
 where an EffectValue significantly different from 1 signifies an impact of X on the EGFR dimerization. Another lysate within the same gel treated identically apart from the exposure of X was used as the reference (Tables 2 and 3).

Exposure of EGF increased dimerization in A431 cells 3.9 times. No effect of EGF on EGFR dimerization was observed in SKOV3 cells.

EGFR dimerization in EGF unexposed A431 cells increased 3.0–3.8 times upon gefitinib tratement (Table 2). Gefitinib increased EGFR dimerization in most of the EGF exposed A431 cells as well, but to a lesser extent (1.4–1.9 times). A significant effect of gefitinib was observed in EGF treated SKOV3 cells (p<0.1), with an increase of 1.8–2.2 times. No effect of gefitinib on EGFR dimerization was detected in EGF untreated SKOV3 cells.

**Table 2:** The effect of gefitinib on EGFR dimerization in A431 and SKOV3 cells.

A431				
	Without pertuzumab		With pertuzumab	
Effect of:	Without EGF	With EGF	Without EGF	With EGF
Gefitinib+complete medium	3.6±1.0*	1.9±0.1*	3.8±0.9*	1.6±0.2*
Gefitinib+serum free medium	3.6±0.8*	1.4±0.1*	3.0±0.6*	1.4±0.2

SKOV3				
	Without pertuzumab		With pertuzumab	
Effect of:	Without EGF	With EGF	Without EGF	With EGF
Gefitinib+complete medium	1.2±0.1*	2.0±0.1*	1.1±0.2	2.1±0.4*
Gefitinib+serum free medium	1.5±0.1*	1.8±0.1*	1.2±0.2	2.2±0.4*

The intensity of the monomer and dimer Western blot bands were analyzed and compared within gels. The table presents EffectValues, i.e. quotients of dimerization degrees. Lysates within the same gel treated identically apart from the exposure of gefitinib (with/without FCS) were used as references. Data are presented as mean±S.E (n = 4).

*indicates treatments affecting dimerization, i.e. with a calculated effect significantly different from 1 (p<0.1). Gefitinib increases dimerization in A431 cells and in EGF treated SKOV3 cells, irrespective of FCS and pertuzumab.

No effect of pertuzumab on the EGFR dimerization in A431 cells was observed (Table 3). In SKOV3 cells, a significant (p<0.1) disruption of in average 40–60% of the EGFR dimerization was detected upon pertuzumab treatment.

**Table 3:** The effect of pertuzumab on EGFR dimerization in A431 and SKOV3 cells.

A431						
	Without EGF			With EGF		
Effect of:	Complete medium	Gefitinib+complete medium	Gefitinib +serum free medium	Complete medium	Gefitinib+complete medium	Gefitinib +serum free medium
Pertuzumab	0.9±0.2	1.1±0.3	1.5±0.1	1.0±0.0	1.5±0.1	1.1±0.1

SKOV3						
	Without EGF			With EGF		
Effect of:	Complete medium	Gefitinib+complete medium	Gefitinib +serum free medium	Complete medium	Gefitinib+complete medium	Gefitinib +serum free medium
Pertuzumab	0.5±0.0*	0.5±0.1*	0.4±0.1*	0.5±0.0*	0.5±0.1*	0.6±0.1*

The table presents EffectValues, i.e. quotients of dimerization degrees. Lysates within the same gel treated identically apart from the exposure of pertuzumab were used as references. Data are presented as mean±S.E (n = 4).

*indicates treatments affecting dimerization, i.e. with a calculated effect significantly different from 1 (p<0.1). Pertuzumab disrupts approximately 50% of the dimerization in SKOV3 cells, regardless of treatment of EGF, FCS or gefitinib. No effects of pertuzumab on EGFR dimerization in A431 cells are observed.

### Lapatinib effects the binding of ^125^I-EGF to A431 cells in a different manner than gefitinib

**Figure 3:**
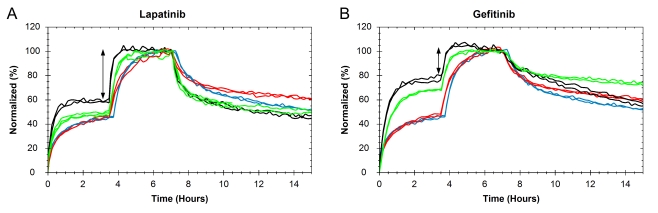
The effect of lapatinib on the ^125^I-EGF–A431 interaction. The binding of ^125^I-EGF to cultured A431 cells treated with complete medium (red), serum free medium (blue), 1 µM of lapatinib (A) or gefitinib (B) in complete medium (green) or 1 µM lapatinib (A) or gefitinib (B) in serum free medium (black) was measured in LigandTracer Grey. A) Lapatinib alters the kinetics of the interaction, as shown as a faster overall association rate and dissociation rate. In serum free medium, the difference between the first and second concentration (indicated by an arrow) is slightly less. B) Gefitinib increases the affinity, observed as a slower dissociation rate and small difference in signal height between the first and second concentration (arrow). The effect of gefitinib is larger in serum free medium.

Exposure of lapatinib modified the kinetics of the ^125^I-EGF–A431 interaction (Fig. 3A). A fast on–fast off interaction was observed in all treatments, but the contribution of it increased when the cells were treated with lapatinib (Fig. 3A, green and black curves). This resulted in an overall faster interaction during both association and dissociation. The difference between the signal height of the first and second concentrations, as indicated by an arrow, is slightly less when treated with serum free medium (Fig. 3A, black curves). These alterations were different from the effects of gefitinib in A431 cells, where the overall affinity increased upon exposure and the dissociation rate varied between complete and serum free medium (Fig. 3B, green and black curves).

### The heterogeneity of the binding of ^125^I-EGF can be visualized by Interaction Maps

*Interaction Map* is a mathematical method to decipher a heterogeneous interaction into its underlying components. It is based on the main assumption that the binding of a ligand to a heterogeneous mixture of targets can be expressed as a sum of interactions [23], [25] where each interaction is represented in an on-off map by its specific pair of recognition (log(k_a_)) and stability (log(k_d_)) coordinates and a color corresponding to the calculated contribution (warm colors–large contribution).

**Figure 4:**
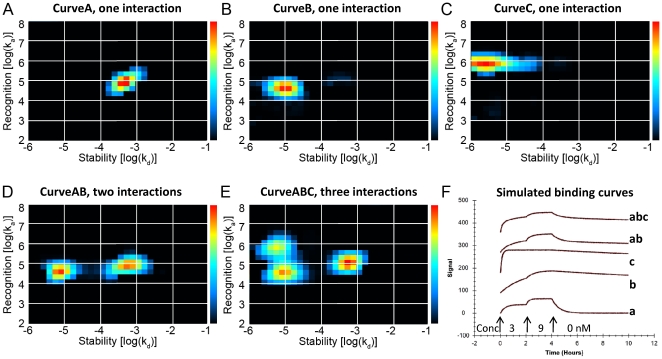
Heterogeneity and kinetic properties of simulated binding curves, presented as Interaction Maps. Interaction Maps were calculated from simulated binding measurements to illustrate the ability to decipher complex interactions. The Interaction Maps display every contributing interaction of the measured binding curve as a peak with specific recognition (log(k_a_)) and stability (log(k_d_)) coordinates using colors representing the relative degree of contribution (red: large contribution, blue: small contribution). Panels A, B, and C contains maps for monovalent interactions (CurveA, CurveB, CurveC). Panel D shows a map for the complex interaction CurveAB known to contain two independent interactions (CurveA+CurveB). Panel E shows a map for the complex interaction CurveABC known to contain three independent interactions (CurveA+CurveB+CurveC). The corresponding simulated binding curves are shown in Panel F. The binder was added first at 3 nM, then at 9 nM, followed by dissociation during several hours, as indicated by the arrows.

**Figure 5:**
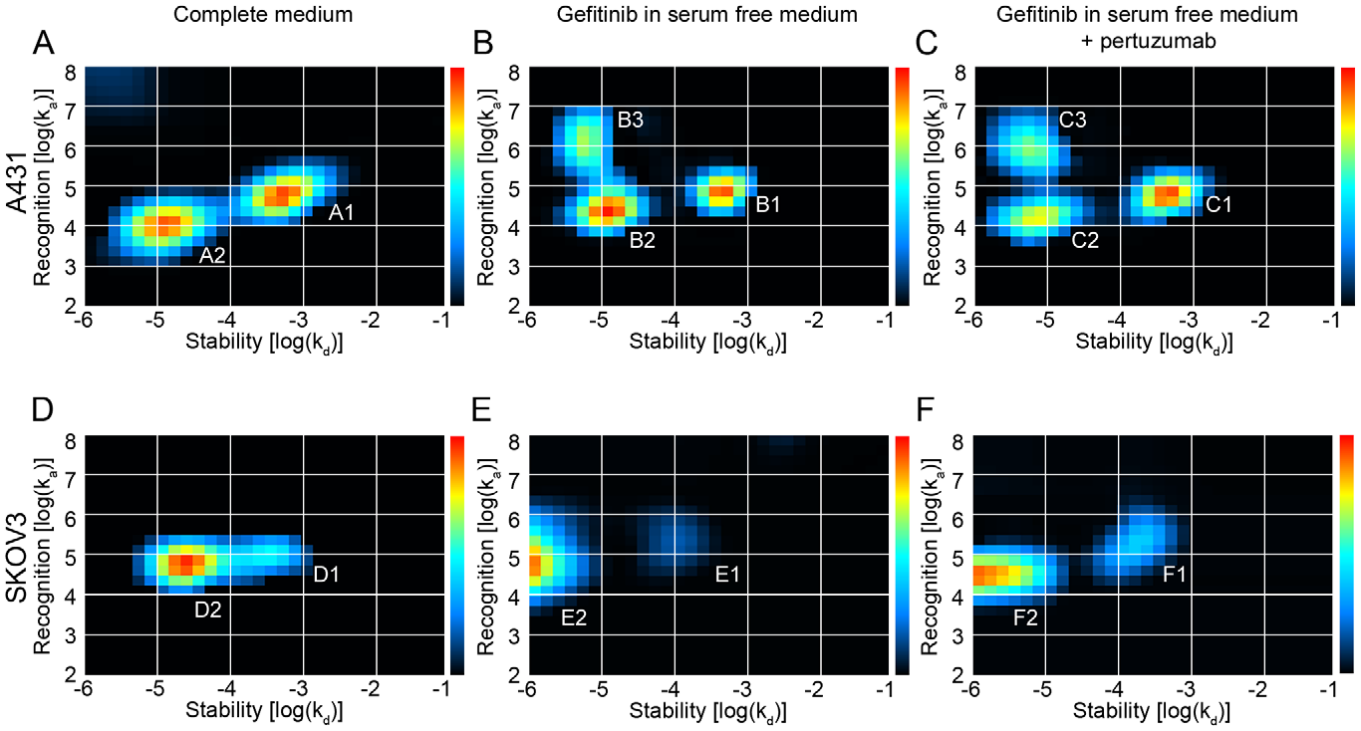
Heterogeneity and kinetic properties of the ^125^I-EGF–EGFR interaction, presented as Interaction Maps. Interaction Maps were calculated from real-time binding measurements of the interaction between ^125^I-EGF and A431 (A–C) or SKOV3 (D–F) cells, using complete medium (A and D), gefitinib in serum free medium (B and E) or gefitinib in serum free medium in the presence of pertuzumab (C and E). A) Under normal conditions in A431 cells, a fast on–fast off (A1: [4.79,−3.25]) and a slow on–slow off (A2: [3.98,−4.94]) interaction are observed. B) When treated with gefitinib in serum free medium, the more stable peak (A2) is slightly shifted to a higher recognition (B2: [4.37,−4.96]), but more importantly another peak at [6.11,−5.28] (B3) appears. C) The coordinates and the contributions of the different peaks are approximately the same with pertuzumab as without, indicating no effect of pertuzumab on the binding of ^125^I-EGF in A431 cells. D) The binding of ^125^I-EGF to SKOV3 cells at normal conditions consists of two interactions at [4.85,−4.59] (D2) and [5.07,−3.48] (D1), the latter less contributing. E) For gefitinib and serum free treated SKOV3 cells, the more stable interaction (D2) is shifted to even higher stability (E2: [4.89,−5.82]) while the contribution of the other interaction is reduced (E1). F) The Interaction Map of gefitinib and serum free treated SKOV3 cells in the presence of pertuzumab seems to be a mixture of Interaction Maps D and E.

Interaction Maps were first calculated on simulated data representing known interaction properties similar to the ones shown to exist in the EGF-EGFR interaction (Fig. 4), which verified that the algorithm could accurately resolve a multitude of different simultaneous interactions. When applied on real interaction data, the Interaction Maps displayed up to three different simultaneous interactions in A431 cells (Fig. 5A–C). The Interaction Map of the binding of ^125^I-EGF to A431 in complete medium depicted two interactions: one fast on–fast off, A1, [4.79, −3.25] with a K_D_ of 9 nM and one slow on–slow off, A2 [3.98,−4.94], with a K_D_ of 1 nM (Fig. 5A). These two interactions contributed approximately equally to the measured binding curve. When exposed to gefitinib in serum free medium (Fig. 5B), the recognition of the more stable interaction increased slightly from log(k_a_): 3.98 (A2) to log(k_a_): 4.37 (B2), while as the weaker interaction (A1) remained in position (B1). Furthermore, a fast on–slow off high affinity interaction (B3) appeared at position [6.11,−5.28], corresponding to a K_D_ of 41 pM. Pertuzumab did not alter the positions and contributions of the peaks (Fig. 5C, peaks C1, C2, C3).

The Interaction Maps of SKOV3 cells presented interaction patterns different to A431 cells. In complete medium two contributing ^125^I-EGF–SKOV3 interactions occurred simultaneously with approximately the same recognition but different stability (D1 [5.07,−3.48] and D2 [4.85,−4.59] (Fig. 5D)). The more stable interaction D2, with a K_D_ of about 0.4 nM, contributed to approximately 65% of the measured binding curve. Upon gefitinib treatment in serum free medium, D2 shifted to a higher stability (E2 [4.89,−5.82]) corresponding to a K_D_ of 20 pM (Fig. 5E). Furthermore, the contribution of the less stable interaction D1 was reduced even further (E1). No equivavelent to the fast on–slow off interaction observed in A431 cells (B3/C3) appeared in gefitinib treated SKOV3 cells. The addition of pertuzumab resulted in an interaction pattern (Fig. 5F) resembling a mixture of the other two Interaction Maps (Fig. 5D and E), with the more stable interaction F2 somewhere in between the previous two positions D2 and E2 while as the weaker interaction F1 remained in position (Fig. 5F).

## Discussion

The aim of this study was to further investigate the binding of ^125^I-EGF to EGFR in order to explain previous results which indicated an impact of the tyrosine kinase inhibitor gefitinib on the interaction [22]. The study started with the quantification of EGFR and HER2 expression on the four previously studied cell lines. The total number of EGFR and HER2 receptors did not vary at different treatments and can thus not explain the effects of gefitinib on the EGF binding.

All EGF incubations described in this paper (i.e. in real-time interaction measurements using LigandTracer and prior to cell lysis for the cross-linking assay) were performed at room temperature. Measurements at 37°C would have been more comparable to the reality inside a body, but the heat triggers EGFR to internalize upon EGF binding, resulting in processing or recycling of EGFR. These intracellular processes are difficult to distinguish from the activities of the membrane bound receptors. Thus, to make the data analysis manageable, room temperature were chosen as an adequate alternative to 37°C. The pre-treatments of gefitinib or pertuzumab before LigandTracer measurements or cell lysis were all done at 37°C.

The antibody pertuzumab disrupts the dimerization ability of HER2, creating fewer EGFR–HER2 heterodimers and more EGFR homodimers [26]. In this paper, real-time interaction data of the binding of ^125^I-EGF to EGFR on A431 and SKOV3 depicts the impact of pertuzumab on association and dissociation rates, thus establishing a connection between dimer quantities and kinetic properties. In A431 and U343 cells, where the HER2 count is about 10% of the EGFR count, no obvious effect of pertuzumab was detected. Upon pertuzumab exposure in HER2 overexpressing SKOV3 cells, the overall affinity decreased in gefitinib treated cells, observed as a higher dissociation rate and larger differences in signal height between the first and second concentration in the binding traces. In other words, pertuzumab seems to interrupt most of the affinity enhancing effect of gefitinib. This indicates that gefitinib may affect the ^125^I-EGF–EGFR interaction in SKOV3 by a process involving dimerization.

A goal in this project was to disrupt EGFR homodimerization as well, in order to investigate its connection to the binding characteristics of the ^125^I-EGF–EGFR interaction. However, no agent was found that accomplish this without simultaneously competing with EGF for the binding site (creating side effects impossible to distinguish from homodimerization disruption effects), thus this could not be established.

By the use of the cross-linking reagent BS^3^, the dimers on the cell surface will remain together during cell lysis and immunoblot analysis [27]. In the cross-linking analysis presented in this paper, EGFR dimers are visible without EGF stimulation in both A431 and SKOV3 cells, however to a low extent in A431 cells. The increase of a factor 4 upon EGF stimulation in A431 cells affirms the hypothesis that EGF binding induces dimerization. On the other hand, no impact of EGF on the EGFR dimerization in SKOV3 cells was detected, providing strength in the hypothesis of existing dimers independent of EGF stimulation. The cell dependent matter of dimerization may be due to differences in EGFR and HER2 receptor quantities. HER2 is known to spontaneously form homodimers [28]–[29]. The findings in this paper indicate that this applies also to EGFR–HER2 heterodimers. EGFR homodimerization on the other hand, appears to require the presence of EGF, as observed in EGFR-rich A431 cells.

In this study, complete medium containing FCS was used in most assays. FCS is known to include some amount of growth stimulators such as EGF, although this is assumed to be low and is not expected to affect the outcome. Furthermore, FCS is often included in similar studies by other groups, making the results comparable.

The immunoblot results further highlight the impact of gefitinib on the dimerization. Gefitinib increased EGFR dimerization in A431 cells, more evidently in the absence of EGF where the overall dimerization was comparably low. In SKOV3 cells on the other hand, the impact of gefitinib was generally less. The induction of EGFR dimers by gefitinib have been studied previously. Yarden et al. proposed that the binding of gefitinib stabilizes an active conformation of the kinase domain of EGFR, creating “quasi-dimers” solely connected intracellularly [21]. Gefitinib is known to prevent growth in both A431 and SKOV3 cells [30], suggesting that the tyrosine kinase activity of the induced EGFR dimers are inhibited, but this does not specify whether they resemble the naturally formed dimers or not. The impact of gefitinib on the kinetics of the extracellular interaction with ^125^I-EGF [22] indicates that the increased dimerization is more than an intracellular phenomenon although further conclusions about the nature of the dimers are difficult to draw.

Serum free medium did not affect the dimerization degree of gefitinib treated cells. This indicates that the effect of serum free medium to the kinetics of the interaction between ^125^I-EGF and gefitinib treated A431 cells cannot be explained by dimerization variation or is too small to be detected by immunoblotting.

The HER2 dimerization inhibitor pertuzumab did not affect the overall EGFR dimerization in A431 cells to a detectable degree, regardless of EGF stimulation, indicating that the EGFR dimers on A431 cells are mostly homodimers. In SKOV3 cells on the other hand, EGFR dimerization drops to 50% upon pertuzumab treatment. The pertuzumab concentration used in the experiment (20 nM) will prevent approximately 75–85% of the HER2 population to form dimers [32], which explains that there are still visible amounts of EGFR dimers in SKOV3 cells. Other factors, like EGFR forming heterodimers with HER3 or HER4 as binding partners are possible but less likely because these receptors are believed to be expressed in much lower levels and are assumed not to affect the outcome much [31]. On the whole, pertuzumab clearly reduces EGFR dimerization.

The combination of pertuzumab and gefitinib is interesting. Looking at the numbers in Table 2 and 3, gefitinib doubles the amount of EGFR dimers in EGF stimulated SKOV3 cells, while pertuzumab simultaneously decreases the amount to 50%. The counteraction of pertuzumab on the gefitinib effect was visible in the real-time interaction data of ^125^I-EGF–SKOV3 as well, yet again suggesting dimerization as a strong contributor to the mechanism of action of gefitinib on the extracellular binding of EGF. As pointed out earlier, it is unclear to what extent EGFR forms heterodimers and homodimers in the cells. Gefitinib may induce solely EGFR homodimers and pertuzumab disrupts EGFR–HER2 dimers but may increase EGFR homodimers. This way, the dimers observed for cells in normal conditions (Fig. 1A, black curve) and when exposed to a combination of gefitinib and pertuzumab (Fig. 1C and D, red curves) may be of different nature, possibly creating the small but visible difference in interaction kinetics observed in the real-time interaction data.

The measurement of the effect of lapatinib on the binding of ^125^I-EGF to A431cells complements the gefitinib study. Lapatinib binds the inactive forms of EGFR and HER2 and does not induce EGFR dimers as opposed to gefitinib that stabilize the active conformation of EGFR [18], [19], [20], [21]. In this paper, real-time interaction data shows that lapatinib affects the binding of ^125^I-EGF, demonstrating that there are other examples of intracellular binders with the potential to influence extracellular interactions. However, the effect of lapatinib on the kinetic properties was unlike what was observed for gefitinib, indicating that the mechanism of action is different between the two tyrosine kinase inhibitors. This strengthens the hypothesis that the generation of EGFR dimers, not observed for lapatinib, is an important part of the effect of gefitinib on the ^125^I-EGF binding. By binding and stabilizing the inactive conformation of EGFR, lapatinib may alternatively inhibit EGFR dimerization, as suggested from the clear, but different, shape of the real-time binding curve.

**Figure 6:**
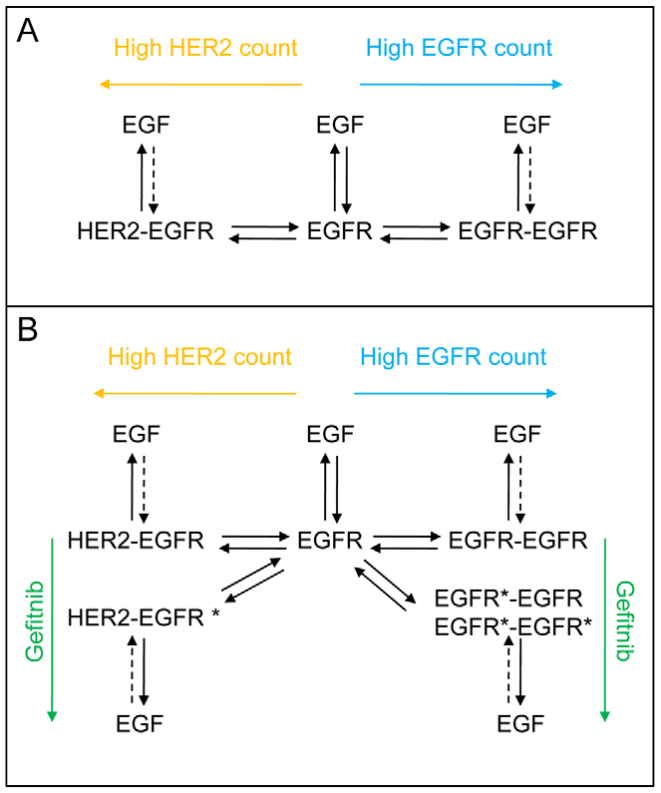
Proposed mechanism of EGF binding to EGFR in normal conditions and when treated with gefitinib. A) Real-time binding data of ^125^I-EGF to A431 and SKOV3 cells, as presented in Interaction Maps, depicts a reality where EGF interacts with not only one receptor form, but several. The different receptor populations may be the monomeric, heterodimeric and homodimeric form of EGFR. The equilibration between the different monomer/dimer states will likely be dependent on the number of EGFR and HER2 receptors expressed on the cell surfaces, where a large HER2 count shifts the equilibrium to more EGFR–HER2 heterodimers and a high EGFR expression results in more EGFR homodimers. Differences in stability of the interaction imply that EGF can dissociate from all three forms. The ability of EGF to bind to ligand free dimers (dashed lines) remains unclear, as the existence of such dimers. B) When treated with gefitinib, the binding of EGF to the dimeric form is altered, suggesting that a new dimeric form is created, either as an addition (A431) or as a replacement (SKOV3).

Interaction Map analysis opens up possibilities for speculation. The two to three visible peaks in each of the Interaction Maps in Figure 5 demonstrate a heterogeneous nature of the binding of EGF to EGFR and provide a detailed description of both the contribution and kinetic properties of the simultaneously occurring interactions. One peak at approximately [5, −3.5] is visible in all six Interaction Maps (A1, B1, C1, D1, E1, F1). A peak representing a more stable interaction is observed as well (A2, B2, C2, D2, E2, F2), but it changes in position and contribution between treatments and cell lines and may even be divided into two peaks in the gefitinib treated A431 cells (B3, C3). One hypothesis is that the right-most peaks (A1–F1) visible in all treatments correspond to the binding of ^125^I-EGF to monomeric EGFR and that the more stable peaks (A2–F2, B3, C3) observed in the left parts of the Interaction Maps represent the interaction between ^125^I-EGF and its two major dimeric forms. The comparably low amount of HER2 receptors in A431 cells makes HER2 an unlikely dimerization partner, leaving EGFR as monomers and homodimers. In SKOV3 cells on the other hand, the large HER2 count will likely “consume” most EGFR receptors, shifting the equilibrium to a more heterodimeric state (Fig. 6A). The differences in A2 and D2 may thus represent the two types of EGFR dimer types. This peak is altered in the presence of the dimerization altering factors gefitinib and pertuzumab, strengthening the idea of it representing the dimeric state. If this hypothesis is valid, Figures 5B and 5E shows that gefitinib induces a shift in contribution from a monomeric state towards a dimeric. It also alters the interaction of EGF with the dimeric form, observed as a new fast on–slow off peak (B3, C3) in A431 cells and a change towards higher stability (from D2 to E2) in SKOV3 cells. The effect of pertuzumab (Fig. 5C and F) indicates that the gefitinib induced dimers are still mostly homodimers in A431 cells (unaffected by pertuzumab) and heterodimers in SKOV3 cells (clearly affected by pertuzumab) (Fig 6B). One necessary experiment for verifying the accuracy of this hypothesis would be to distinguish between EGFR homodimers and EGFR–HER2 heterodimers, in normal conditions and upon gefitinib treatment. Gefitinib is directed towards EGFR [33], suggesting that the induced dimers are solely EGFR homodimers if presuming the need of gefitinib binding to both binding partners for a functioning dimerization. However, kinase inhibitors are notoriously promiscuous [34] and it is possible that gefitinib also binds to HER2 to some extent.

The question remains whether EGF can bind to pre-existing EGFR dimers or if the differences in interaction are merely the result of EGF dissociating from either EGFR monomers or dimers. The observed association rate of a ligand to its target will relate to the recognition and stability of the interaction and the concentration of the ligand, while the observed dissociation rate is dependent on the stability alone [35]. The differences in stability between the observed peaks can thus represent various forms of dissociation. In A431 cells there are differences in recognition as well, indicating that EGF is able to not only dissociate from, but also associate *to* at least two different structures. Our hypothesis is that these structures are EGFR monomers and EGFR dimers, i.e. ^125^I-EGF binds independently and directly to both EGFR monomers and existing EGFR homo/hetero dimers on A431 cells.

One of the goals of this paper was to establish the biological processes behind the effect of the tyrosine kinase inhibitor gefitinib on the EGF–EGFR interaction. We propose that gefitinib induces dimers with higher affinity to ^125^I-EGF than EGFR monomers or the EGFR dimers normally displayed on the cell surface. Furthermore, the heterogeneity of the ^125^I-EGF–EGFR interaction proposes an explanation to the previously observed differences in affinity between cell lines. The epidermal growth factor receptors may be identical in both sequence and tertiary structure, but to what extent they form dimers may depend on e.g. the receptor expression of the hosting cell line. Each EGFR monomer/dimer form interacts with EGF with specific kinetics properties (recognition and stability), which results in differences in overall affinity depending on the prevalence of these interactions. Interaction Maps can decipher a measured interaction into its contributing components, thus opens up for a detailed understanding of the EGFR family puzzle.

In conclusion, the results presented in this paper show that gefitinib induces formation of EGFR dimers which alters the apparent interaction characteristics with EGF. The prevalence of monomers, homodimers and heterodimers are different between cell lines and are likely dependent on EGFR and HER2 expression. EGF binds to the various monomeric/dimeric forms of EGFR with unique binding properties, thus the distribution of the different EGFR forms in a cell will affect the overall affinity.

## Materials and Methods

### Antibodies and reagents

The tyrosine kinase inhibitors gefitinib and lapatinib was from Biaffin GmbH (Kassel, Germany) and GlaxoSmithKline (London, UK) respectively. Cetuximab was purified from Erbitux® (Merck KGaA, Darmstadt, Germany) and trastuzumab was purified from Herceptin® (Roche AB, Stockholm, Sweden). The humanized monoclonal antibody pertuzumab, directed towards HER2 to prevent dimerization, was purified from Omnitarg™ from Genentech (San Fransisco, CA, USA). The cross-linking reagent bis(sulfosuccinimidyl)suberate (BS^3^) was purchased from Thermo Scientific (Rockford, IL, USA). The polyclonal rabbit anti-EGFR antibody was from Santa Cruz (Santa Cruz, CA, USA) and the monoclonal mouse anti-β-actin antibody (clone AC-15) was from Sigma Aldrich (St Louis, MO, USA). Peroxidase-conjugated anti-rabbit antibody and peroxidase-conjugated anti-mouse antibody was purchased from GE Healthcare (Waukesha, WI, USA).

### Cell culture

Four cell lines were used: the human squamous carcinoma cell line A431 (CLR 1555, ATCC, Rocksville, MD, USA), the human ovarian carcinoma cell line SKOV3 (HTB-77, ATCC, Rocksville, MD, USA) the human glioma cell line U343MGaCl2:6 (a subclone of U343MG [36]), denoted U343, and the human breast cancer cell line SKBR3 (HTB-30, ATCC, Rocksville, MD, USA). In all cellular experiments, the cells were grown in a humified incubator at 37°C, equilibrated with 5% C0_2_ until experimental day. The cells were cultured in Ham's F10 cell culture medium (Biochrom Ag, Berlin, Germany) supplemented with 10% fetal calf serum (Sigma, St Louis, MO, USA), L-glutamine (2 mM) and PEST (penicillin 100 IU/ml and streptomycin 100 µg/ml, from Biochrom Ag, Berlin Germany) if not otherwise specified.

### Radiolabeling

Human Epidermal growth factor (EGF, Chemicon International, USA) was labeled with ^125^I (Perkin-Elmer, Wellesley, MA, USA) using the Chloramine-T protocol [37].

### Treatment of cells with tyrosine kinase inhibitors

Cells were exposed to four different treatments, as described previously [22]. In brief, cells were incubated for 24−48 h in either complete or serum free Haḿs F10 culturing medium in the presence or absence of 1 µM of either gefitinib or lapatinib.

### Estimating number of receptors

The expression levels of EGFR and HER2 were estimated using either the manual or the kinetic extrapolation (KEX) method, as described by Bárta et. al. [24]. For the manual protocol, cells were seeded in triplicates in 12-wells plates (NuclonTM, Roskilde, Denmark). ^125^I-labeled cetuximab and trastuzumab were used for the quantification of EGFR and HER2 respectively. A single concentration of 100 nM antibody was estimated to be sufficient for saturating the receptors, as it corresponds to approximately 100×K_D_. After 3–4 hours of incubation at 4 °C, the cells were washed repeatedly with serum free medium, followed by trypsination. The number of resuspended cells were counted and the radioactivity measured (automatic gamma counter 1480 WIZARD™ 3”, PerkinElmer) to evaluate cell receptor levels [38]. For the KEX method, cells were incubated with increasing concentrations of either ^125^I-labeled trastuzumab or cetuximab in LigandTracer Grey at room temperature. The binding traces were to a kinetic model describing a bivalent interaction, using TraceDrawer 1.2 (Ridgeview Instruments AB, Uppsala, Sweden). This resulted in an estimation of B_max_, which was used to correct for non-saturation of receptors.

### Real-time interaction measurements of ^125^EGF in LigandTracer

Real-time measurements of the binding of ^125^I-EGF to EGFR on the cells were performed at room temperature in LigandTracer Grey instruments, according to previously published protocol [39]. After a short baseline, the cells were incubated for 3.5 h twice using increasing concentrations of ^125^I-EGF. The concentrations were chosen based on the affinity to EGF for the three cell lines (2.8 and 9 nM for A431, 0.5 and 1.5 nM for U343, 0.7 and 2 nM for SKOV3). The ligand solution was replaced with fresh medium (containing 1 µM gefitinib or 1 µM lapatinib in the TKI treatment studies) and the dissociation rate was followed over night.

### Dimerization perturbation

To prevent EGFR-HER2 dimerization, A431, SKOV3 and U343 cells were incubated with 20 nM of pertuzumab over night prior to the detection of the ^125^I-EGF – EGFR interaction in LigandTracer Grey or the cross-linking experiments. The antibody remained in the ^125^I-EGF solution during measurement (LigandTracer) or EGF incubation (cross-linking). Pertuzumab effects were studied in combination with four of the treatments described above (complete medium, serum free medium, 1 µM gefitinib and serum free medium+gefitinib).

### Cross-linking of EGFR and HER2

Prior to cross-linking, the cells were treated with or without 1 µM gefitinib (48 h) and 20 nM pertuzumab (over-night) in 37°C as described previously, followed by incubation with or without EGF (9 nM) for 3.5 hours at room temperature. After three times washing with ice cold PBS, the cells were subjected to 1 mM of the cross-linking reagent BS^3^at room temperature for 30 min. The reaction was terminated using 20 mM (final concentration) Tris-Cl, pH 8.5 for 15 min at room temperature. Subsequently, cells were washed with ice cold PBS and solubillized in lysis buffer containing 150 mM NaCl, 20 mM Tris(amino), 5 mM EDTA, 10% (v/v) glycerol and 1% (v/v) Triton X-100 and a cocktail of protease and phosphatase inhibitors.

### Immunoblotting and image analysis

Cell lysates were separated by SDS-PAGE in a 3–8% tris-acetate gel (Invitrogen, Carlsbad, CA, USA) and transferred to PVDF membranes (Millipore Corporation, Billerica, MA, USA), which were blocked for 1 h with PBS-T with 5% BSA (w/v) before incubation with primary antibodies at 4°C over-nigh. The proteins were visualized using peroxidase-conjugated ImmobilionTM Western Chemiluminescent HRP Substrate (Millipore Corporation, Billerica, MA, USA) in a cooled charge-coupled device (CCD) camera (FujiFilm, Luminescent Image analyzer LAS-1000plus). The intensity of bands was quantified in ImageJ (Rasband, W.S., ImageJ, U. S. National Institutes of Health, Bethesda, Maryland, USA, http://imagej.nih.gov/ij/, 1997–2011) to study the consequences of treatments upon dimerization. Statistical analysis was performed in Minitab® 15 (Minitab Inc., State College, PA, USA) using Student's t-tests. Results with p-values less than 0.1 were deemed significant. The HiMark™ (Invitrogen, Carlsbad, CA, USA) molecular weight protein standards ranging from 31 kDa to 460 kDa was used to assess the molecular weight of identified bands.

### Interaction Map analysis

The mathematical method Interaction Map [25] expresses the measured binding of a (homogeneous) ligand to a heterogeneous group of targets as a sum of interactions, each having a unique combination of the association rate constant k_a_ and dissociation rate constant k_d_:



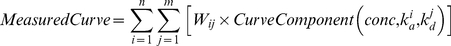
where conc is the concentration of protein in solution (the “ligand”) and W_ij_ is the weighing factor describing the contribution to the measured real-time interaction curve. The calculated contributing curves are represented as colored (red = large W_ij_, dark blue = small W_ij_) peaks in an on-off plot. In this paper, the Interaction Map method used 24 (k_a_)×30 (k_d_) different nodes with kinetic parameter values evenly distributed in log-space (log10(k_a_) = {2.0000, 2.2609, 2.5217, …, 7.4783, 7.7391, 8.0000} , log10(k_d_) = {-6.0000, −5.8276, −5.6552, …, −1.3448, −1.1724, −1.0000}). A Tichonov-type regularization algorithm was used to real-time binding curves measured in LigandTracer Grey, adding penalty to the sum-of-square residuals if there are many peaks in the Interaction Map. A similar algorithm has been presented previously [23] and has been applied to SPR-based real-time interaction analysis.

The non-linear fitting algorithm was implemented in Visual Basic/Visual Studio 2003 (Microsoft, Mountain View, CA). The ability of the Interaction Map algorithm to accurately decompose a complex binding data was validated on a series of binding curves simulated in MATLAB 6.5 (The Mathworks, Natic, MA, USA). The peak position was estimated as the center of gravity for the peak and is reported as [log10(k_a_) , log10(k_d_)]. The contribution of a peak was estimated as the percentage of the weight factors (W_ij_) for the pixels of the peak in comparison to the sum of all weight factors.

The interaction map algorithm was tested using a range of different simulated binding curves with known interaction properties. In this paper, simulated curves with similar binding properties as what was found in the investigated ^125^I-EGF - EGFR interaction were used: monovalent CurveA (k_a_ = 10^5^ M^−1^s^−1^; k_d_ = 5*10^−4^ s^−1^), monovalent CurveB (k_a_ = 5*10^4^ M^−1^s^−1^; k_d_ = 10^−5^ s^−1^), monovalent CurveC (k_a_ = 8*10^5^ M^−1^s^−1^; k_d_ = 8*10^−6^ s^−1^), complex CurveAB (CurveA+CurveB) and complex CurveABC (CurveA+CurveB+CurveC). In all cases, an assay of first 2 h of 3 nM binder, then 2 h of 9 nM binder and finally 6 h of dissociation (i.e. 0 nM binder) was simulated. Noise corresponding to 1% of the signal was added to each curve. These five curves were subjected to Interaction Map analysis and were expected to produce single, double or triple peak maps.
